# Validation of the Arabic Version of Diabetes Eating Problem Survey–Revised (DEPS-R) among Adolescents with Type 1 Diabetes

**DOI:** 10.3390/nu15030561

**Published:** 2023-01-21

**Authors:** Abdulrahman Hummadi, Saeed Yafei, Mohammed Badedi, Raed Abutaleb, Hussain Darraj, Ali Jaber Alhagawy, Abdullah Khawaji, Yahia Solan, Turki Alzughbi, Mohammed Hakami, Sattam Jaddoh, Abdulrraheem Daghriri, Mamdouh Khardali

**Affiliations:** 1 Jazan Endocrinology & Diabetes Center, Ministry of Health, Jazan 45142, Saudi Arabia; 2 Faculty of Medicine and Health Sciences, Taiz University, Taiz P.O. Box 6803, Yemen; 3 Administration of Research & Studies, Jazan Health Affairs, Jazan 82611, Saudi Arabia; 4 Psychology Department, Jazan Endocrinology & Diabetes Center, Jazan 82723, Saudi Arabia; 5 Jazan Psychiatry Hospital, Ministry of Health, Jazan 45142, Saudi Arabia

**Keywords:** disordered eating behaviors, diabetes eating problem survey–revised, DEPS-R, eating attitudes test, Arabic, type 1 diabetes

## Abstract

Disordered eating behaviors (DEBs) in type 1 diabetes (T1D) have been studied globally in different age groups. However, there is no validated diabetes-specific questionnaire in the Arabic language for the screening of DEBs. This study aimed to translate the Diabetes Eating Problem Survey–Revised scale (DEPS-R) into the Arabic language and study its psychometric properties in adolescents with T1D. We adopted the forward–backward procedure to translate the DEPS-R questionnaire into Arabic. The validation study enrolled 258 Saudi adolescents with T1D aged 12–18 years who completed the Arabic DEPS-R questionnaire and the EAT-26. The Arabic DEPS-R showed good construct validity and reliability (Cronbach’s alpha = 0.86). The factor analysis revealed a three-factor structure of DEPS-R which explains 54.4 % of the variance. In total, 30.6% of the participants are at high risk for DEBs (DEPS-R score ≥ 20). The psychometric properties of the Arabic DEPS-R are satisfactory, consistent with the original scale and translations in other languages. These results support the validity of the Arabic DEPS-R for assessment of DEBs in the T1D Arabic population.

## 1. Introduction

The Arab world consists of 22 Arab-speaking countries with high rates of consanguinity, shared lifestyles and eating habits. According to the Diabetes Atlas, 2021, about 1.2 million people under the age of 20 have type 1 diabetes (T1D) worldwide [1]. Saudi Arabia, Algeria and Morocco are the three Arab countries featured at the top 10 countries with the highest prevalence and incidence of T1D among children and adolescents below the age of 20 [1].

Eating disorders (EDs) include anorexia nervosa and bulimia nervosa in addition to other specified or unspecified EDs. These four categories have definite diagnostic criteria in the ICD-10 or DSM-5 and are characterized by preoccupation with food, body weight and shape [2]. Milder eating problems were recognized as subclinical eating disorders or disordered eating behaviors (DEBs) [3]. Individuals with DEBs frequently experience eating disorder symptoms, but they do not match the requirements for diagnosis of eating disorder syndromes [4]. 

In the Arab countries, it was anticipated that ED prevalence would be low. In recent years, however, the prevalence of EDs and DEBs is increasing, especially in females [5]. According to a recent review, [6] the prevalence of DEBs ranges from 13% to 55%, with variations among Arab countries. The high prevalence of diabetes and obesity, sociocultural changes, Westernization, globalization, the impact of the media, urbanization and an increase in the popularity of the thin ideal are all identified triggers for ED in the Arab world [7]. 

T1D is a form of diabetes which developed as a consequence of autoimmune destruction of the beta cells of the pancreas, resulting in absolute insulin deficiency. T1D is mostly diagnosed in childhood or adolescence; however, it might be diagnosed in adults. Until now, insulin therapy is the only available treatment for people with T1D [8]. There is general agreement that adolescents with T1D are more susceptible to disordered eating, but the prevalence differed across the studies depending on sex, age range and measures used [3,9]. According to the available literature, adolescent females, especially, are at the highest risk for DEBs and EDs [3,4]. A systematic review study found that adolescents with T1D have a two- to three-fold higher prevalence of DEBs than non-diabetic individuals of similar age and sex [3]. Furthermore, a number of studies in T1D patients revealed significant association between DEBs and poor glycemic control [10,11]. Other studies found that T1D people with eating problems are at higher risk for diabetes-related complications and higher mortality rates [12,13]. 

Clinical interview is the gold standard for EDs diagnosis, but in most circumstances, it might be difficult and time-consuming to conduct a clinical interview. Hence, screening tools for DEBs were developed where people who scored above a clinical cut-off on these screening measures are considered at higher risk for the more pathologic syndromes of eating disorders [4]. For the screening of eating behaviors, different screening tools are well established, including the Eating Attitudes Test (EAT-26), Eating Disorder Examination Questionnaire (EDE-Q) and SCOFF, in addition to others [9]. However, the use of these traditional tools in people with T1D produces a diagnostic challenge. Diabetes patients usually have diabetes-specific eating behaviors such as insulin omission or restriction, which cannot be assessed by the general tools [9]. For this reason, specific instruments were developed for investigation of DEBs in diabetes, such as the Diabetes Eating Problem Survey–Revised (DEPS-R), modified Sick, Control, One Stone, Fat, Food (mSCOFF) and the Screen for Early Eating Disorder Signs (SEEDS) [9].

The DEPS-R is a diabetes-specific screening tool for DEBs, developed in English by Markowitz et al. [14]. It is considered as the best validated tool for DEBs in children, adolescents and adults [9]. Recently, DEPS-R is recommended for screening of DEBs in youth with T1D by the American Diabetes Association (ADA) and the IPSAD [15,16]. The original DEPS-R composed of 16 items, each item with 6 responses on a 6-point Likert scale. The overall DEPS-R score ranges from zero to 80, so people who have higher DEPS-R total scores are at greater risk of EDs. According to the original version of DEPS-R, a total score at or above 20 was established as a threshold point indicating greater eating pathology [14].

DEPS-R has been translated and validated among T1D and T2D patients at different ages in different countries, such as Germany [17], Norway [18], Turkey [19], Spain [20], Italy [21], Canada [22], China [23] and Greece [24]. However, there is no validated Arabic version of the DEPS-R. Thus, the aim of this study was to translate the DEPS-R scale into the Arabic language and study its psychometric properties among adolescents with T1D.

## 2. Methods

### 2.1. Design, Setting and Permission

This study was conducted in two steps: the translation process and the cross-sectional validation of the DEPS-R. The translation of DEPS-R followed the forward–backward procedure for translation of scales [25]. This study was conducted at Jazan Endocrinology & Diabetes Center (JEDC), Saudi Arabia, from March to July 2022. JEDC is a high-volume multidisciplinary endocrine center in Saudi Arabia highly equipped to manage people with diabetes. Permission for the Arabic translation and validation of the original DEPS-R scale for this study was provided by the developer of the English version of DEPS-R, Prof. Dr. Lisa Volkening, Associate Director of Pediatric Research Programs at Joslin Diabetes Center.

### 2.2. Forward Translation

Three Arabic native translators completed the forward translation of the original English version of DEPS-R into the Arabic language independently, and one of the translators was a bilingual diabetologist. 

Expert committee included the translators, a diabetologist, a pediatric endocrinologist, an independent Arabic professor of the English language, and a bilingual psychologist experienced in diabetes. The expert committee meeting compared the Arabic drafts to each other as well as the original English version and discussed the most accurate terms. All items were revised and the most appropriate Arabic phrasing was agreed upon, thus producing a single forward translation of DEPS-R (Arabic draft).

### 2.3. Back-Translation

Back translation to English was performed by two bilingual translators, independent from the previous translators and unaware of the purpose of the scale and forward translation technique. The back-translations were reviewed by the expert committee to compare the semantic equivalence and resolve any uncertainties and differences in the meanings of words or sentences. They compared the Arabic draft, the original scale and the back-translation producing the final Arabic DEPS-R. No item was deleted or added, so the final Arabic version of DEPS-R included the same 16 items as in the original version.

### 2.4. Participants and Measures

#### 2.4.1. Participants

The study population included adolescents aged 12 to 18 years with T1D who are being followed at JEDC, Saudi Arabia. For the validation studies, at least 10 participants are needed for each scale item [26]. Since the DEPS-R scale included 16 items, the minimal required sample was 160. As this study is a cross-sectional which also evaluates the prevalence of DEBs among T1D, we calculated the sample size using the Epi-info Stat-Calc program so the final sample included 258 adolescent males and females. From the patient registry, participants were selected randomly using an electronic number generator. Participants were restricted to include Saudi adolescents diagnosed with T1D for at least 1 year and on multiple daily insulin therapy (MDI). Exclusion criteria included type 2 diabetes patients or specific types of diabetes other than type 1 diabetes, patients with very high HbA1c and patients with mental or physical disabilities, serious diabetes complications or other medical conditions such as celiac disease, hemoglobinopathies and uncontrolled thyroid diseases. The selected participants were contacted on the day of their regular follow-up visits.

Sociodemographic data, including age, gender, weight, height, education level, duration of T1D and treatment modality were recorded in a specific sheet. Clinical, anthropometric and biochemical data were retrieved from the medical files. Body mass index (BMI) (kg/m^2^) was calculated as weight (kg) over height (m) and adjusted for age and sex. Glycemic data included the fasting plasma glucose, HbA1c, total daily insulin dose and other results recorded from continuous glucose monitors (CGM).

#### 2.4.2. DEPS-R

After clinical evaluation, participants completed the Arabic DEPS-R questionnaire in a paper-based model with a diabetes nurse or a dietitian who clarified any item that needed more understanding. Items were scored according to the original scale on a six-point Likert scale where “0” represents “never,” and “5” represents “always.” DEPS-R score cutoff point ≥ 20 indicates a high risk for DEBs. The overall score ranges from zero to 80, with a higher score indicating more DEBs [14]. 

#### 2.4.3. EAT-26 

Participants also completed the EAT-26, which was used with permission obtained from the developer of the test [27]. This test has been extensively used as a standardized, self-report traditional scale for the screening of subjects at risk of DEBs. EAT-26 is arranged in three sub-scales: Diet, Bulimia and Food Control, and it was validated previously in an Arabic-speaking population [28]. In the Arabic version of the questionnaire a cutoff point at or higher than 20 was established to indicate the risk of DEBs in the general population [28].

### 2.5. Psychometric Properties

Psychometric properties in this study included evaluation for the different types of reliability and validity. Content validity refers to the degree in which the instrument content adequately reflects the construct that is being measured [29,30]. It can be assessed qualitatively by an expert committee and then qualitatively by using the content validity index (CVI) [25,29]. In this study, we assessed the content validity by the expert committee with the calculation of scale-CVI [31].

Criterion validity is the relation between the score of a certain instrument and some external criterion [29]. It can be assessed through comparison with results obtained from an established-criterion intended to measure the same criterion [30]. For this purpose, we assessed the concurrent correlation between the Arabic DEPS-R total score and the EAT-26 total score. Furthermore, the correlation between DEPS-R scores, HbA1c and BMI was calculated using Pearson’s correlation coefficients. 

Construct validity is the degree to which a group of variables really represents the construct to be measured [29]. To evaluate the factorial validity of the Arabic DEPS-R, we conducted an exploratory factor analysis (EFA) using the principal component analysis with Kaiser normalization and varimax rotation. Bartlett’s and Kaiser–Meyer–Olkin (KMO) tests were used to evaluate the suitability of the data for factor analysis. Factor loadings more than 0.40 were considered sufficient to include the item. 

For reliability assessment, we evaluated the stability and internal consistency. Stability was assessed by the test/retest method on a subgroup of the participants (*n* = 64) who completed the test twice within a 4-week interval. We calculated the intraclass correlation coefficient (ICC) to evaluate the stability. Cronbach’s alpha coefficient was estimated to assess the internal consistency for the whole sample. 

### 2.6. Statistical Analysis

Statistical analysis was conducted using SPSS Version 26 (IBM, Armonk, NY, USA). Level of significance was determined at *p* < 0.05. Normality of distribution of variables was determined visually and by the Shapiro–Wilk test. Continuous variables with normal distributions were expressed as mean ± standard deviations (SD), while medians represented other variables. For comparison between continuous variables, the independent t-test or the Mann–Whitney test were used as indicated. The correlation between DEPS-R score and other variables was assessed using Pearson’s coefficient. 

## 3. Results

### 3.1. Participant Characteristics

The current study recruited 258 male and female adolescents. Females represent 62.8% of the total sample (162/258). The mean age was 15.6 ± 1.8 years (12 to 18 years). The mean duration for T1D was 6.7 ± 3.9 years (range 1–15) and the mean age of diagnosis was 8.9 ± 3.6 years. The mean BMI of the participants was 21.2 ± 4.6 kg/m^2^. The baseline characteristics and comparison of the main clinical parameters between males and females are summarized in Table 1.

### 3.2. DEPS-R Scores

Most of the participants completed the Arabic DEPS-R within 5 min, which is a reasonable time when used in clinical practice. The mean DEPS-R total was 14.8 ± 10.9, and the median DEPS-R score was 13 (range 0 to 46). Approximately 30.6% (79/258) of the participants scored ≥ 20 on the Arabic DEPS-R score. Prevalence of DEBs in females was 36.4% compared to 20.8% of males (*p* = 0.009). Females had a significantly higher DEPS-R scores of 16 ± 11.5 compared to 12.9 ± 9.8 in males (*p* = 0.023). On the EAT-26, only 18.2% of the participants scored ≥ 20.

### 3.3. Content Validity

The back-translated version of the Arabic DEPS-R showed overall semantic similarities to the items in the original scale. A copy of the back-translated version was sent back to the developer of the original scale at Joslin who approved the translation. Furthermore, the Arabic DEPS-R was assessed by seven experts including two endocrinologists, two family physicians, two diabetes nurses and a psychologist. We calculated the scale content validity index (S-CVI) to assess whether the Arabic DEPS-R scale subjectively addresses the concept of DEBs in diabetes patients. Experts were asked to evaluate each item by scoring between 1 and 4 (not appropriate, needs major revision, needs minor revision, appropriate). The overall S-CVI of this scale was 0.91, which signifies the adequacy of this scale to achieve its purpose.

### 3.4. Criterion and Construct Validity

In this study, we investigated the presence of individuals at high risk of ED using two scales, the EAT-26 and the DEPS-R. We found that 18.2% of the participants are at high risk of ED (47/258) and scored ≥ 20 on the EAT-26. However, in the DEPS-R score, 30.6% of the total sample scored positive for DEBs (79/258). Furthermore, 63.8% of those who scored positive on the EAT-26 were also positive in DEPS-R (30/47). So, DEPS-R detected more positive DEBs cases than those detected by the EAT-26 scale. This indicates that participants who are at high risk of eating disorders on the general scale were also at high risk according to the specific DEPS-R questionnaire. Concurrent validity was established also by the significant correlation of the Arabic DEPS-R total score with the EAT-26 scale (r, 0.49; *p* < 0.001), HbA1c (r, 0.57, *p* < 0.001) and BMI (r, 0.41; *p* < 0.001). DEPS-R has weak correlation with age and diabetes duration. Correlations of DEPS-R with other variables in males and females are provided in Table 2.

### 3.5. Exploratory Factor Analysis

All items in the original DEPS-R scale were included in the factorial analysis as there was no over-correlation between items (>0.9), no low communality (<0.3) and no item with low-loading on factors (<0.4). The KMO coefficient for the Arabic-DEPS-R was 0.86, and the Bartlett’s test of sphericity analysis was *p* < 0.001.

The EFA revealed three factors with eigenvalues > 1, which accounts for a total variance explained of 54.4% (Table 3). On the basis of eigenvalues and inspection of the scree plot, three factors were selected for further assessment with varimax rotation. The first factor included 9 items related to eating habits (items 2, 3, 4, 5, 7, 12, 13, 14 and 15)and accounted for 33.9% of the variance. The second factor included 4 items (items 1, 6, 11 and 16) related to the preoccupation with thinness and explaining 11.8% of the variance. Factor 3 included 3 items (item 8, 9, 10) concerned with desire to maintain high blood glucose and explained 8.7% of the variance. All three factors showed relatively strong loadings and items loaded exclusively on one component (0.51–0.82).

### 3.6. Reliability

The Arabic DEPS-R showed good internal consistency in this group of adolescents with T1D, where the Cronbach’s alpha coefficient of the total sample was 0.86 (95% CI 0.83–0.88); Cronbach’s α was 0.86 in the female group (95% CI 0.83–0.89), and in the male group it was 0.84 (95% CI 0.79–0.88). The corrected item-to-total-score correlations were more than 0.31, supporting the internal consistency of the scale. We assessed the temporal stability by the test/retest reliability and intraclass correlation coefficient was calculated. Therefore, the ICC was 0.89, which is satisfactory (95% CI: 0.77–0.95, *p* < 0.001).

## 4. Discussion

The current study is the first validation study which evaluates the psychometric properties of the Arabic version of the DEPS-R in Saudi adolescents with T1D. According to the results obtained, the Arabic DEPS-R demonstrates satisfactory psychometric properties. It demonstrates good internal consistency; Cronbach’s alpha coefficient of the total sample was 0.86. This is consistent with the original DEPS-R validation [14] and also consistent with the results obtained in Norway, Italy, China and Turkey [17,19,21,23]. The small variation in Cronbach’s α values in different studies could be explained in part by the fact that the Cronbach’s α is not a characteristic for the entire test but rather for the sample used in the test [29]. Adolescents with T1D share several characteristics, including eating attitudes, self-esteem and similar worries, so their response to the same scale could give comparable results. The ICC was 0.89, which indicates good temporal stability of this questionnaire. 

The original DEPS-R was primarily developed as a single-factor scale [14]. On further validation it was organized into three factors named maladaptive eating, preoccupation with thinness and maintaining high blood glucose to lose weight [17]. In this study, EFA of the Arabic DEPS-R items revealed 3 factors explaining 54.4% of the variance with acceptable factor loadings of items (0.51–0.82). This three-factor pattern in the Arabic DEPS-R is comparable with previous validation studies conducted in Norway [32], China [23] and Italy [23]. 

The Spanish validation of the DEPS-R [20] in an adult population yielded five factors with cumulative variance of 55.28%, referred to as food attitudes, bulimic behaviors, weight control, avoidance, and restriction. Another EFA study in Greek [33] adults revealed four factors (diet, weight loss, insulin use and compensatory behaviors) explaining 65.68% of the total variance, but it included a small sample of 100 participants. Recently, another Greek study on 100 adults using confirmatory factor analysis supported a single-factor model among T1D adult patients [24]. 

Our findings support the previous validations of DEPS-R [14,17,18,20], where the Arabic DEPS-R showed good criterion and construct validity. It has an acceptable positive correlation with EAT-26, indicating the effectiveness of the Arabic DEPS-R questionnaire compared to the EAT-26 (r = 0.49, *p* < 0.001). DEPS-R was compared with different general screening tools of DEBs in different countries. In the original scale, Markowitz et al. [14] reported positive correlation with the eating subscale of the Diabetes Quality of Life for Youth (DQOLY) (r = 0.59) in US adolescents. A Norwegian study [17] indicated a significant positive correlation between DEPS-R and EAT-12 (r = 0.65). According to a German study [18], DEPS-R displayed good construct validity and had significant correlation with SCOFF and the Eating Disorder Examination Questionnaire–EDE-Q, (r = 0.54 and 0.70, respectively). The Greek validation study of DEPS-R on 100 adults demonstrated good construct validity and significant correlation with the EAT-26 (r = 0.38) [33]. In a Spanish study, DEPS-R was also significantly correlated with the EAT-26 in adults (r = 0.44) [20]. These results are not different to the results obtained from the current study and altogether indicate the suitability of DEPS-R for rapid screening of DEBs in T1D. 

Concurrent validity in this study is established also through the significant correlations of the Arabic DEPS-R total score with HbA1c and BMI. Participants who scored > 20 have higher HbA1c values. That is in line with the original scale, in which the DEPS-R score showed significant correlations with age, BMI and HbA1c [14]. Similar findings were obtained also in different studies which reported that people with high DEPS-R have higher BMI and higher HbA1c and were at greater risk for eating disorders [18,22,34]. Furthermore, we found that when compared to EAT-26, the DEPS-R was more strongly correlated with HbA1c. Similar results were also obtained in a study comparing DEPS-R and EAT-12 [17]. 

Overall, our results showed that 30.6% of the sample had DEPS-R scores ≥20, and more females scored at or above 20 compared to males (36.4% vs. 20.8%, *p* = 0.009). DEPS-R mean scores ± SD in the present study were 14.8 ± 10.9 in the total sample, with 12.9 ± 9.8 in males and 16 ± 11.5 in females. These findings are not so different from of other studies with some variations according to age and sex. For example, Cherubini et al. [34] reported 34.4% of T1D adolescents have DEBs (41.7% of females vs. 26.6% of males). Nip et al. [35] reported 21.2% of T1D adolescents have DEBs (30% of females vs. 12% of males). However, Wisting et al. [17] found that only 18.3% of 770 children and adolescents have DEBs, with higher prevalence in females (27.7% in females vs. 8.6% in males). Furthermore, the total DEPS-R score was significantly higher in females compared to males in different studies [17,18,19]. 

The observed gender differences in DEB prevalence and DEPS-R score in different studies are consistent in the existing literature [2]. These differences cannot be explained only by the higher number of females selected in these studies, but other factors may exist. Adolescent females with T1DM have more risk factors for DEBs, including more dietary restraint, greater body dissatisfaction and more diabetes-specific negative effects, more weight gain in females and more frequent meal skipping, so they are at higher risk for EDs [2]. 

This study was not without limitations. Firstly, the study did not include the gold standard interview for assessment of EDs so we could not estimate the sensitivity and specificity of the test. Furthermore, the present study did not include people above the age of 18 and people with very high HbA1c or those with complications or other disease, so the results may be different in these groups, especially more prevalence of insulin restrictions or omission. Future work may further seek to examine confirmatory factor analysis and to use clinical interview and EDs diagnostic criteria to search the true positive cases of EDs and to start psychotherapy and investigate its effectiveness on glycemic control.

## 5. Conclusions

This study outlines the Arabic translation and psychometric properties of DEPS-R in Saudi adolescents with T1D. Psychometric properties of the Arabic DEPS-R are satisfactory and consistent with the original scale and the translations in other languages. These results support the usefulness of the Arabic DEPS-R for assessment of DEBs in people with type 1 diabetes.

## Figures and Tables

**Table 1:** Basic characteristics of the participants with comparison of males and females.

Variables	Total *n* = 258	Female *n* = 162	Male *n* = 96	Female vs. Male, *p* Value
**Age (years)**	15.6 ± 1.8	15.7 ±1.9	15.3 ± 1.8	0.053
**Duration (years)**	6.7 ± 3.9	7 ± 3.8	6 ± 4	0.051
**Diabetes onset (years)**	8.9 ± 3.6	8.7 ± 3.5	9.2 ± 3.9	0.280
**BMI (kg/m^2^)**	21.2 ± 4.6	21.7 ± 4.4	20.3 ± 4.6	0.010 *
**HbA1c (%)**	8.7 ± 1.8	8.8 ± 1.8	8.4 ± 1.8	0.151
**Insulin dose (U/kg/day)**	1.1 ± 0.4	1.1 ± 0.4	1.1 ± 0.4	0.567
**DEPS-R score**	14.8 ± 10.9	16 ± 11.5	12.9 ± 9.8	0.023 *
**DEPS-R score ≥ 20, *n* (%)**	79 (30.6)	59 (36.4)	20 (20.8)	0.009 *
**EAT26 score**	13.1 ± 8.5	13.5 ± 8.6	12.4 ± 8.4	0.320
**EAT-26 score ≥ 20, *n* (%)**	47 (18.2)	32 (19.8)	15 (15.6)	0.406

Data are presented as mean ± SD, unless otherwise specified, with *p*-values for the significance of the difference between females and males; * *p* is significant at the 0.05 level.

**Table 2:** Correlations of DEPS-R total score to other variables.

Variables	Total r, *p*	Female r, *p*	Male r, *p*
**EAT-26**	0.49, <0.001 *	0.53, <0.001 *	0.38, <0.001 *
**Age**	0.26, <0.001 *	0.23, 0.003 *	0.28, 0.006 *
**BMI**	0.41, <0.001 *	0.44, <0.001 *	0.31, 0.002 *
**HbA1c**	0.57, <0.001 *	0.60, <0.001 *	0.50, <0.001 *
**Duration of diabetes**	0.15, 0.017 *	0.16, 0.043 *	0.09, 0.392

r, correlation coefficient; * *p* is significant at the 0.05 level.

**Table 3:** Exploratory factor analysis with rotated component matrix *.

Item	Factor 1	Factor 2	Factor 3
Other people have told me that my eating is out of control	0.721		
I feel that my eating is out of control	0.685		
I skip meals and/or snacks	0.673		
I alternate between eating very little and eating huge amounts	0.669		
I eat more when I am alone than when I am with others	0.636		
When I overeat, I don’t take enough insulin to cover the food	0.635		
Other people tell me to take better care of my diabetes	0.619	0.301	
I avoid checking my blood sugar when I feel like it is out of range.	0.536		
After I overeat, I skip my next insulin dose	0.507		
Losing weight is an important goal to me		0.829	
I would rather be thin than have good control of my diabetes		0.739	
I feel that it’s difficult to lose weight and control my diabetes at the same time		0.725	
I feel fat when I take all of my insulin		0.666	
I try to eat to the point of spilling ketones in my urine.			0.817
I try to keep my blood sugar high so that I will lose weight			0.812
I make myself vomit			0.810
**Percent of Variance explained, %**	33.9	11.8	8.7
**Eigenvalues**	5.416	1.883	1.397

* Principal components with varimax rotation and Kaiser normalization.

## Data Availability

The data presented in this study are available on reasonable request from the corresponding author.
